# Changes in Renal Resistive Index Values in Healthy Puppies during the First Months of Life

**DOI:** 10.3390/ani10081338

**Published:** 2020-08-03

**Authors:** Amalia Agut, Marta Soler, M. Josefa Fernández-del Palacio

**Affiliations:** Department of Animal Medicine and Surgery, Veterinary Faculty, University of Murcia, 30100 Murcia, Spain; mtasoler@um.es (M.S.); mjfp@um.es (M.J.F.-d.P.)

**Keywords:** Doppler ultrasound, resistive index, kidney, intrarenal artery, dog, puppy

## Abstract

**Simple Summary:**

The renal resistive index (RRI) is probably the most commonly used parameter to evaluate blood flow in kidney vessels. Alterations in this parameter have been noted in a range of conditions affecting the kidney, such as acute variations in renal vascular resistance and renal damage in multiple-organ dysfunction syndrome. In order to interpret changes of the RRI in pathologic conditions, the normal values of intrarenal arterial RI should be known. In adult healthy dogs, the RRI values have been well established. However, there is little information regarding the RRI values of puppies. The objective of this study is to establish the RRI of normal kidneys in puppies aged from newborn to 20 weeks of age and to determine the age at which RRI reaches adult dog values. The highest value was obtained in the first three weeks of life and then it gradually declined with increasing age reaching adult dog values at 12 weeks of age.

**Abstract:**

The purpose of this study is to establish renal resistive index (RRI) of normal kidneys in puppies aged from newborn to 20 weeks of age and to determine the age at which RRI reaches adult dog values. Six healthy adult intact beagles and six puppies from 1 day after birth to 20 weeks of age were used. In the adult dogs, the ultrasonographic scans were performed once, and in the puppies, the ultrasonographic studies were performed on the first day after birth and at 1, 2, 3, 4, 6, 8, 12, 16, and 20 weeks of age. RRI was obtained at the interlobular and arcuate arteries in each kidney. There were no statistical differences between the RRI values obtained between the right and left kidney nor between intrarenal arteries (interlobar and arcuate). The RRI was the highest during the first weeks of life, after which it declined gradually with increasing age reaching adult dog values at 12 weeks of age. In conclusion, the normal mean RRI is age dependent in dogs. Twelve weeks can be regarded as the age at which adult mean RRI criteria can be applied to puppies.

## 1. Introduction

Renal blood flow is closely linked to physiological and pathological changes in the kidney [1]. Doppler ultrasound can be used to assess the renal blood flow. Several parameters have been used to evaluate changes in the renal vasculature and blood flow [2]. The renal resistive index (RRI) is probably the most commonly used parameter to evaluate blood flow in kidney vessels [2,3]. The resistive index (RI) measures the arterial resistance in the peripheral vessels by calculating the ratio between the peak systolic velocity (PSV) and the end diastolic velocity (EDV) (RI = (PSV – EDV)/PSV), which is independent of the angle and the position of the transducer, allowing accurate and reproducible measurements [4]. This is particularly important for the evaluation of glomerular, tubulointerstitial, and vascular lesions in the kidney [5,6]. Alterations in this parameter have been noted in a range of conditions affecting the kidney, such as acute variations in renal vascular resistance and renal damage in multiple-organ dysfunction syndrome [5,6].

Both in humans [7,8] and dogs [9], it has been demonstrated that RRI is age dependent. However, little information regarding intrarenal arterial RI in puppies is available. In order to interpret changes of the RRI in pathologic conditions in puppies, the normal values of intrarenal arterial RI should be established.

The purpose of this study is to establish the RRI of normal kidneys in puppies aged from newborn to 20 weeks of age and to determine the age at which RRI reaches adult dog values.

## 2. Materials and Methods

This study was approved by the Ethical Committee of the University of Murcia (616/20). All procedures were carried out in accordance with the current Spanish (RD 53/2013) and European (Directive 2010/63/EU) legislation on animal protection.

### 2.1. Animals

The present study was carried out with six healthy adult intact beagles (3 males and 3 females) with a mean age of 4 years (range, 2 to 6 years) and mean body weight of 10.7 kg (range, 8 to 13 kg), which were previously used in ultrasound practical lectures, and six beagle puppies (2 males and 4 females) of different litters which were evaluated from 1 day after birth to 20 weeks of age. The study was extended to 20 weeks of age since in a previous study [9] the renal RI in dogs younger than 4 months of age was higher than in older dogs. The puppies were the progeny of a closed Beagle colony at the Animal Medicine and Surgery Department, University of Murcia.

Each dog was housed individually in facilities in accordance with animal care and use guidelines. The puppies were housed with their mother and allowed free access to them after weaning.

All the adult animals were determined to be clinically healthy on the basis of results of physical examination, complete blood cell counts, serum biochemical profiles, urine analyses, urine protein-to-creatinine ratio, and abdominal ultrasound. During the first 4 weeks of life, the puppies were considered healthy based on their behavior, physical examination, urinalysis, and abdominal ultrasound for evaluating the appearance of both kidneys. The complete blood cell counts, and serum biochemical profiles were performed at 4 weeks of age.

### 2.2. Ultrasonographic Evaluations

The ultrasonographic scans in the adult beagles (control group) were performed once. In the puppies, the ultrasonographic studies were performed on the first day after birth and at 1, 2, 3, 4, 6, 8, 12, 16, and 20 weeks of age.

All ultrasonographic examinations were performed using 5–8 MHz curved array transducer (HD15, Philips, Eindhoven, The Netherlands). The researcher adapted the settings to obtain the best image quality for visualizing the kidney in each dog. The dogs were examined without sedation. They were manually restrained in lateral recumbency, which varied according to scanned kidney. The hair was clipped, and acoustic coupling gel was applied to the skin over the area of interest in each dog when it was necessary. All the abdominal ultrasound examinations were performed by the same board-certified radiologist (A.A.).

B-mode, color Doppler, and pulsed wave Doppler ultrasound examinations were performed on both kidneys to assess the RI of the intrarenal arteries. Each kidney was imagined in the longitudinal plane in B-mode and then color flow Doppler ultrasonography was utilized to visualize the intrarenal vasculature. The interlobar arteries were localized along the border of medullary pyramids and the arcuate arteries at the corticomedullary junctions [6]. Then, pulse-wave Doppler ultrasonography was performed for each of the intrarenal arteries with a Doppler sample width of 1–2 mm. The lowest possible filter was used and the smallest scale that displayed the flow without aliasing was selected. Three to five similar sequential Doppler waveforms were obtained. Then the measurement of the RI was calculated automatically by the software of the ultrasonographic machine after manual delimitation of the PSV and EDV using the internal calipers. RI at the interlobular and arcuate arteries was measured in three separates sites within each kidney and the average value was calculated.

### 2.3. Statistical Analysis

The sample size was calculated using the “resource equation method” [10].

Data editing and statistical analyses were performed using the IBM SPSS v24.0 (SPSS Inc., Chicago, IL, USA). Normality of variable distribution was tested by the Shapiro–Wilk test. The Mann–Whitney test was used to assess differences in the RI values between the right and left kidney, and between interlobar and arcuate arteries. The effect of age in the renal RI values was analyzed with the Kruskal–Wallis test. The Wilcoxon matched pairs signed rank test was used to compare differences in RI values between the different ages of the puppies. The Mann–Whitney U test was used to explore differences in RI values between puppies and adult dogs. Data are shown as mean ± standard deviation (SD) (in the text and table) and median together with percentiles (in the figures). The results were considered statistically significant when *p* ≤ 0.05.

## 3. Results

All the data obtained in the study are available within the article and its Supplementary Materials.

Both kidneys were identified in each dog and presented normal structure and greyscale ultrasonographic appearance. Doppler examinations were successfully performed in all animals. Although, in puppies, the Doppler flow spectrum of arcuate arteries was more difficult to obtain until six weeks age.

No significant differences were found in the RI values obtained between the interlobular (0.73 ± 0.1) and arcuate (0.75 ± 0.13) arteries. There were no significant differences in the RI values obtained between the right (0.75 ± 0.11) and left (0.74 ± 0.12) kidneys. Accordingly, the results are focused on the effect of age on RRI values.

The mean of RRI values obtained in puppies and adult dogs is presented in Table 1.

**Figure 1:**
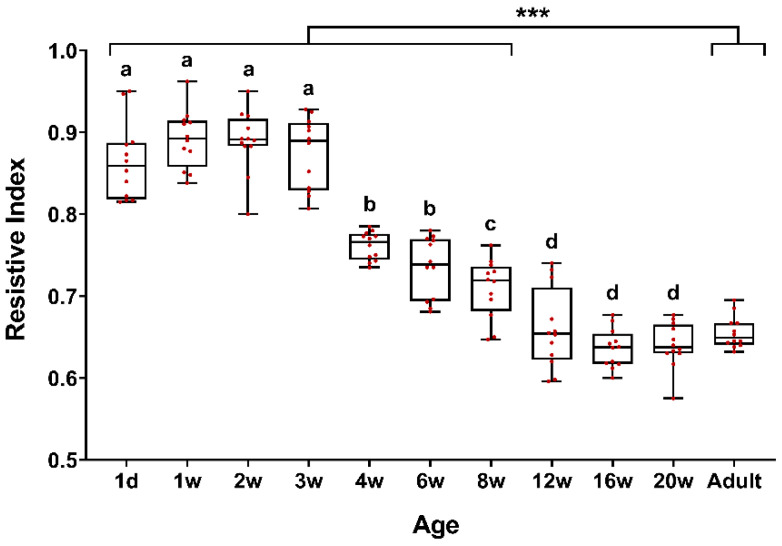
Box-whisker plot showing the renal resistive index (RRI) over the age of the puppies and the adult dogs. Horizontal lines in the boxes represent the median, the bottom, and top of the boxes, the 25th and 75th percentiles; and the whiskers indicate the minimum and maximum values. The red points indicate the RRI value of each dog for each kidney. *** Indicates differences (*p* ≤ 0.05) between the ages of puppies and adult dogs. a–d: Indicates differences (*p* ≤ 0.05) between the puppies’ ages.

**Figure 2:**
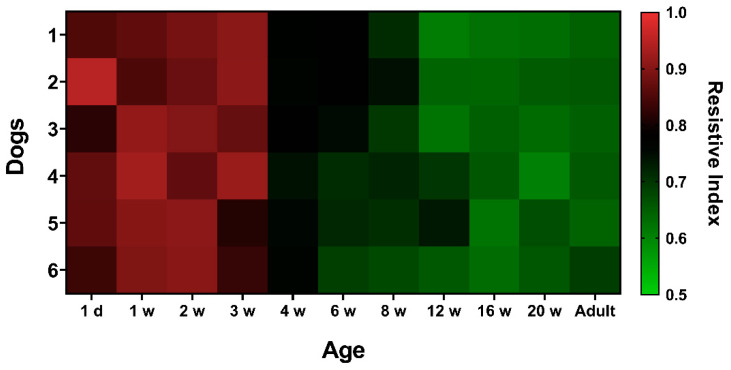
Heat map showing the renal resistive index value for each dog over the different ages of the puppies and the adult dogs. Red shows the high values and green the low values of the resistive index.

Age influenced the RRI values (Figure 1). The RRI values tended to decrease with increasing age. An alternative form of data presentation is a color-coded heatmap according to RRI values (Figure 2). Renal RI values were significantly higher from birth to three weeks of age, and then gradually decreased stabilizing at 12 weeks of age, where they reached the adult dog values.

There were no significant differences between the RRI values obtained from puppies from 1 day after birth to three weeks of age (Figure 1). However, there were differences (*p* ≤ 0.05) between the RRI values obtained at these ages and the rest of the ages of puppies and adult dogs. The RRI values obtained at four weeks and six weeks of age were similar. There were differences (*p* ≤ 0.05) between them and the values obtained at the different ages of puppies and adult dogs. There was a difference (*p* ≤ 0.05) between the RRI values obtained at eight weeks of age and the rest of the ages of puppies and adult dogs. There were no differences (*p* ≤ 0.05) between the RRI values of the puppies from 12 weeks of age and adult dogs.

## 4. Discussion

This is the first study, to the authors’ knowledge, that shows the evolution of the RRI in dogs from birth to 20 weeks of age. There was no statistical difference between RI values obtained between the right and left kidney nor between intrarenal arteries (interlobar and arcuate). The RRI was the highest at birth and then gradually decreased with increasing age reaching adult dog values at 12 weeks of age.

Intrarenal RI values did not vary between the right and left kidney. This finding is in agreement with the renal arteries of humans [8,11], canines [1], and felines [1,12]. In contrast, Tipisca et al. [4] reported significantly higher RI values in the left than right kidneys in healthy cats. In horses, the RI in the right kidney was statistically higher than in the left kidney [13]. These authors reported that different RI values between both kidneys is difficult to explain. Unless there were any microscopical differences in functional subunits which are not known, or technical factors, since ultrasonography is essentially an operator-dependent method [4,13].

No differences were found between RRI values obtained in interlobar and arcuate arteries [7,9]. However, in puppies during the first weeks of age, the Doppler flow spectrum for arcuate arteries was more difficult to obtain due to low blood flow to the kidney cortex in neonates [14]. The cortical flow increased with age as a result of both an increase in cortical size and perfusion per gram of tissue [14]. Nevertheless, it has been suggested that many pathologic processes induce the most marked alterations in resistance to blood flow in the most distal arterial branches within the kidney. Thus, Doppler flow spectra obtained from arcuate arteries would potentially have greater clinical relevance for measurements in renal disease [6].

The intrarenal RI values showed a significant dependence on age [9,15]. In humans, intrarenal RI values are highest at birth and decline with age [7,15]. In veterinary medicine, there is only a study where the RI values of dogs younger than four months were compared with dogs older than four months. The RI on dogs <4 months of age was higher than in older dogs [9]. However, to the authors’ knowledge, repeated assessment of RRI from birth in the same dog has not been reported. In addition, a precise definition of when the transition from puppies to adult RRI values takes place remains to be answered.

In our study, as in previous human studies [7,8], intrarenal RI values were the highest at birth and then gradually declined with increasing age. The mean value of RRI in the newborns was 0.86. This value remained until three weeks of age. Then, they gradually decreased from four to 12 weeks of age and remained stable from 12 weeks of age, reaching the RRI values obtained in the adult dogs (0.66 ± 0.02). Chang et al. [9] reported a mean RI of 0.75 ± 0.05 in dogs <4 months. These values are close to those obtained in our study for the puppies from four to eight weeks of age but are higher than in older puppies from 12 to 20 weeks of age. In the above-mentioned report [9], the authors obtained RRI values of 0.65 ± 0.05 for the dogs >4 months. These values are similar to those obtained in our study for puppies from 12 weeks of age and the adult dogs, being within the reported reference values (0.56–0.67) [1,16].

These findings should be explained due to developmental physiology and renal anatomy in dogs. The puppy kidney is immature in both structure and function at birth. Functional maturity of the kidney lags behind anatomic maturation [17]. The nephrogenesis in dogs continues for at least two weeks after birth [18]. Until approximately eight days of age, there is a distinct subcapsular zone in which new nephrons are formed. Beneath this nephrogenic zone, the nephrons (specifically the glomeruli) become increasingly more mature towards the corticomedullary junction. Although it is common to detect fetal or immature glomeruli until 21 days of age, these should decrease in number after that time [19]. During this period, the RRI values were the highest, caused by the large number of immature glomeruli [15]. After nephrogenesis has ceased, the growth and maturation of the kidney continues for a long time [19]. At 74 days old, the time glomerular function, expressed per unit of renal weight reached the mature level. Moreover, the microscopic appearance of kidneys of 70-day-old dogs is similar to that of adult dogs. The glomeruli are mature and there is less difference in size between glomeruli of the outer and inner cortex [19]. These features support our findings where the RRI values remained stable from 12 weeks of age, reaching the RI values obtained in the adult dogs. Hence, the decline in renal vascular resistance with age reflects the process of ongoing maturation of the kidney after birth [7].

Another explanation could be that the kidneys’ volume increases with age causing an increase in vascular diameter and cross-sectional area, then leading a decrease of RI [15]. The appearance of the vascular system of the puppy kidney is strikingly different from the adult dog kidney. From birth to six weeks of age, the mean renal blood flow increases progressively, reaching 60–70% of the total blood flow at about 8–10 weeks of age and the adult value of blood flow at 14–16 weeks of age [14]. These features could also explain that at 12 weeks of age the RRI values of adult dogs are reached.

Other reports have suggested that the progressive decline of RI values with age is related to active plasma renin levels [7,9].

Perhaps the main limitation of this study is the small sample size. However, we have used as few animals as possible to obtain sufficient data to achieve the objectives of this study. Since the animals of this investigation were not able to be replaced by other techniques or methods. Another limitation could be that all the examinations and measurements were performed by the same operator. Ultrasound is operator dependent, therefore variations that could occur between operators were not evaluated.

## 5. Conclusions

The normal mean RI is age dependent in dogs. The highest value was obtained in the first three weeks of life and then it gradually declined with increasing age reaching adult dog values at 12 weeks of age.

## Figures and Tables

**Table 1:** Renal resistive index values obtained at different ages of puppies and adult dogs.

Age	Mean ± SD	Range
1 day	0.86 ± 0.05	0.81–0.95
1 w	0.89 ± 0.03	0.84–0.96
2 w	0.89 ± 0.04	0.8–0.95
3 w	0.87 ± 0.04	0.81–0.93
4 w	0.76 ± 0.02	0.73–0.78
6 w	0.73 ± 0.04	0.68–0.78
8 w	0.71 ± 0.04	0.65–0.76
12 w	0.66 ± 0.05	0.6–0.74
16 w	0.64 ± 0.02	0.6–0.68
20 w	0.64 ± 0.03	0.57–0.68
Adult	0.66 ± 0.02	0.63–0.69

w: week.

## Supplementary Materials

The following are available online at https://www.mdpi.com/2076-2615/10/8/1338/s1, Material S1: Data of RRI puppies.
