# Supplementary material for: Changes in Renal Resistive Index Values in Healthy Puppies during the First Months of Life
Source: Animals (Basel). 2020 Aug 3;10(8):1338. doi: 10.3390/ani10081338 (PMC7459847; doi:10.3390/ani10081338)
Supplement: Supplementary file 1 [file animals-10-01338-s001.zip › supplementary material.pdf]

Age	Dogs	Kidney	Artery	RI
1	1	1	1	0,81
1	1	1	1	0,8
1	1	1	1	0,9
1	1	2	1	0,83
1	1	2	1	0,82
1	1	2	1	0,82
1	1	1	2	0,89
1	1	1	2	0,95
1	1	1	2	0,96
1	1	2	2	0,8
1	1	2	2	0,82
1	1	2	2	0,81
1	2	1	1	0,94
1	2	1	1	0,95
1	2	1	1	0,96
1	2	2	1	0,94
1	2	2	1	0,96
1	2	2	1	0,94
1	2	1	2	0,96
1	2	1	2	0,96
1	2	1	2	0,96
1	2	2	2	0,97
1	2	2	2	0,96
1	2	2	2	0,97
1	3	1	1	0,8
1	3	1	1	0,8
1	3	1	1	0,8
1	3	2	1	0,85
1	3	2	1	0,84
1	3	2	1	0,83
1	3	1	2	0,82
1	3	1	2	0,83
1	3	1	2	0,84
1	3	2	2	0,79
1	3	2	2	0,8
1	3	2	2	0,82
1	4	1	1	0,86
1	4	1	1	0,85
1	4	1	1	0,87
1	4	2	1	0,86
1	4	2	1	0,89
1	4	2	1	0,87
1	4	1	2	0,85
1	4	1	2	0,87
1	4	1	2	0,89
1	4	2	2	0,85
1	4	2	2	0,89

1	4	2	2	0,88
1	5	1	1	0,95
1	5	1	1	0,87
1	5	1	1	0,8
1	5	2	1	0,85
1	5	2	1	0,87
1	5	2	1	0,88
1	5	1	2	0,79
1	5	1	2	0,8
1	5	1	2	0,83
1	5	2	2	0,92
1	5	2	2	0,9
1	5	2	2	0,91
1	6	1	1	0,81
1	6	1	1	0,8
1	6	1	1	0,8
1	6	2	1	0,83
1	6	2	1	0,82
1	6	2	1	0,82
1	6	1	2	0,89
1	6	1	2	0,92
1	6	1	2	0,9
1	6	2	2	0,8
1	6	2	2	0,82
1	6	2	2	0,81
2	1	1	1	0,86
2	1	1	1	0,87
2	1	1	1	0,87
2	1	2	1	0,82
2	1	2	1	0,84
2	1	2	1	0,87
2	1	1	2	0,89
2	1	1	2	0,9
2	1	1	2	0,95
2	1	2	2	0,84
2	1	2	2	0,82
2	1	2	2	0,84
2	2	1	1	0,8
2	2	1	1	0,8
2	2	1	1	0,8
2	2	2	1	0,8
2	2	2	1	0,8
2	2	2	1	0,8
2	2	1	2	0,81
2	2	1	2	0,8
2	2	1	2	0,82
2	2	2	2	0,86
2	2	2	2	0,82

2	2	2	2	0,8
2	3	1	1	0,84
2	3	1	1	0,85
2	3	1	1	0,88
2	3	2	1	0,88
2	3	2	1	0,87
2	3	2	1	0,87
2	3	1	2	0,96
2	3	1	2	0,97
2	3	1	2	0,96
2	3	2	2	0,97
2	3	2	2	0,97
2	3	2	2	0,93
2	4	1	1	0,82
2	4	1	1	0,82
2	4	1	1	0,83
2	4	2	1	0,98
2	4	2	1	0,97
2	4	2	1	0,96
2	4	1	2	0,96
2	4	1	2	0,97
2	4	1	2	0,97
2	4	2	2	0,96
2	4	2	2	0,95
2	4	2	2	0,95
2	5	1	1	0,8
2	5	1	1	0,8
2	5	1	1	0,87
2	5	2	1	0,88
2	5	2	1	0,88
2	5	2	1	0,85
2	5	1	2	0,9
2	5	1	2	0,96
2	5	1	2	0,95
2	5	2	2	0,97
2	5	2	2	0,97
2	5	2	2	0,97
2	6	1	1	0,86
2	6	1	1	0,86
2	6	1	1	0,87
2	6	2	1	0,78
2	6	2	1	0,79
2	6	2	1	0,79
2	6	1	2	0,97
2	6	1	2	0,96
2	6	1	2	0,95
2	6	2	2	0,97
2	6	2	2	0,98

2	6	2	2	0,95
3	1	1	1	0,87
3	1	1	1	0,87
3	1	1	1	0,87
3	1	2	1	0,82
3	1	2	1	0,8
3	1	2	1	0,79
3	1	1	2	0,91
3	1	1	2	0,92
3	1	1	2	0,9
3	1	2	2	0,96
3	1	2	2	0,97
3	1	2	2	0,96
3	2	1	1	0,98
3	2	1	1	0,97
3	2	1	1	0,98
3	2	2	1	0,78
3	2	2	1	0,8
3	2	2	1	0,79
3	2	1	2	0,96
3	2	1	2	0,96
3	2	1	2	0,92
3	2	2	2	0,97
3	2	2	2	0,97
3	2	2	2	0,97
3	3	1	1	0,82
3	3	1	1	0,86
3	3	1	1	0,85
3	3	2	1	0,85
3	3	2	1	0,81
3	3	2	1	0,84
3	3	1	2	0,98
3	3	1	2	0,96
3	3	1	2	0,96
3	3	2	2	0,95
3	3	2	2	0,97
3	3	2	2	0,93
3	4	1	1	0,83
3	4	1	1	0,89
3	4	1	1	0,89
3	4	2	1	0,82
3	4	2	1	0,83
3	4	2	1	0,85
3	4	1	2	0,82
3	4	1	2	0,83
3	4	1	2	0,81
3	4	2	2	0,95
3	4	2	2	0,94

3	4	2	2	0,93
3	5	1	1	0,91
3	5	1	1	0,89
3	5	1	1	0,9
3	5	2	1	0,85
3	5	2	1	0,86
3	5	2	1	0,85
3	5	1	2	0,95
3	5	1	2	0,94
3	5	1	2	0,93
3	5	2	2	0,92
3	5	2	2	0,95
3	5	2	2	0,92
3	6	1	1	0,89
3	6	1	1	0,91
3	6	1	1	0,89
3	6	2	1	0,83
3	6	2	1	0,82
3	6	2	1	0,8
3	6	1	2	0,95
3	6	1	2	0,94
3	6	1	2	0,95
3	6	2	2	0,95
3	6	2	2	0,95
3	6	2	2	0,95
4	1	1	1	0,85
4	1	1	1	0,86
4	1	1	1	0,84
4	1	2	1	0,84
4	1	2	1	0,85
4	1	2	1	0,83
4	1	1	2	0,97
4	1	1	2	0,96
4	1	1	2	0,96
4	1	2	2	0,97
4	1	2	2	0,97
4	1	2	2	0,95
4	2	1	1	0,85
4	2	1	1	0,84
4	2	1	1	0,8
4	2	2	1	0,95
4	2	2	1	0,94
4	2	2	1	0,95
4	2	1	2	0,92
4	2	1	2	0,95
4	2	1	2	0,96
4	2	2	2	0,97
4	2	2	2	0,9

4	2	2	2	0,86
4	3	1	1	0,84
4	3	1	1	0,82
4	3	1	1	0,84
4	3	2	1	0,83
4	3	2	1	0,8
4	3	2	1	0,82
4	3	1	2	0,88
4	3	1	2	0,88
4	3	1	2	0,85
4	3	2	2	0,97
4	3	2	2	0,97
4	3	2	2	0,96
4	4	1	1	0,88
4	4	1	1	0,9
4	4	1	1	0,89
4	4	2	1	0,79
4	4	2	1	0,75
4	4	2	1	0,77
4	4	1	2	0,96
4	4	1	2	0,96
4	4	1	2	0,96
4	4	2	2	0,95
4	4	2	2	0,97
4	4	2	2	0,96
4	5	1	1	0,82
4	5	1	1	0,81
4	5	1	1	0,84
4	5	2	1	0,8
4	5	2	1	0,8
4	5	2	1	0,79
4	5	1	2	0,8
4	5	1	2	0,83
4	5	1	2	0,83
4	5	2	2	0,8
4	5	2	2	0,82
4	5	2	2	0,83
4	6	1	1	0,85
4	6	1	1	0,83
4	6	1	1	0,84
4	6	2	1	0,83
4	6	2	1	0,84
4	6	2	1	0,85
4	6	1	2	0,82
4	6	1	2	0,83
4	6	1	2	0,82
4	6	2	2	0,8
4	6	2	2	0,82

4	6	2	2	0,83
5	1	1	1	0,78
5	1	1	1	0,74
5	1	1	1	0,78
5	1	2	1	0,75
5	1	2	1	0,77
5	1	2	1	0,79
5	1	1	2	0,78
5	1	1	2	0,79
5	1	1	2	0,77
5	1	2	2	0,77
5	1	2	2	0,74
5	1	2	2	0,75
5	2	1	1	0,74
5	2	1	1	0,76
5	2	1	1	0,77
5	2	2	1	0,8
5	2	2	1	0,78
5	2	2	1	0,78
5	2	1	2	0,76
5	2	1	2	0,75
5	2	1	2	0,72
5	2	2	2	0,76
5	2	2	2	0,77
5	2	2	2	0,75
5	3	1	1	0,77
5	3	1	1	0,73
5	3	1	1	0,75
5	3	2	1	0,8
5	3	2	1	0,79
5	3	2	1	0,78
5	3	1	2	0,83
5	3	1	2	0,82
5	3	1	2	0,81
5	3	2	2	0,77
5	3	2	2	0,75
5	3	2	2	0,73
5	4	1	1	0,75
5	4	1	1	0,74
5	4	1	1	0,7
5	4	2	1	0,8
5	4	2	1	0,77
5	4	2	1	0,78
5	4	1	2	0,72
5	4	1	2	0,77
5	4	1	2	0,76
5	4	2	2	0,72
5	4	2	2	0,7

5	4	2	2	0,72
5	5	1	1	0,78
5	5	1	1	0,8
5	5	1	1	0,8
5	5	2	1	0,7
5	5	2	1	0,75
5	5	2	1	0,71
5	5	1	2	0,82
5	5	1	2	0,72
5	5	1	2	0,74
5	5	2	2	0,76
5	5	2	2	0,75
5	5	2	2	0,74
5	6	1	1	0,76
5	6	1	1	0,76
5	6	1	1	0,78
5	6	2	1	0,8
5	6	2	1	0,78
5	6	2	1	0,79
5	6	1	2	0,74
5	6	1	2	0,7
5	6	1	2	0,72
5	6	2	2	0,78
5	6	2	2	0,77
5	6	2	2	0,76
6	1	1	1	0,76
6	1	1	1	0,76
6	1	1	1	0,78
6	1	2	1	0,77
6	1	2	1	0,78
6	1	2	1	0,78
6	1	1	2	0,78
6	1	1	2	0,76
6	1	1	2	0,74
6	1	2	2	0,79
6	1	2	2	0,78
6	1	2	2	0,78
6	2	1	1	0,8
6	2	1	1	0,79
6	2	1	1	0,8
6	2	2	1	0,78
6	2	2	1	0,78
6	2	2	1	0,75
6	2	1	2	0,77
6	2	1	2	0,74
6	2	1	2	0,74
6	2	2	2	0,76
6	2	2	2	0,77

6	2	2	2	0,78
6	3	1	1	0,78
6	3	1	1	0,74
6	3	1	1	0,78
6	3	2	1	0,79
6	3	2	1	0,77
6	3	2	1	0,74
6	3	1	2	0,79
6	3	1	2	0,77
6	3	1	2	0,75
6	3	2	2	0,71
6	3	2	2	0,7
6	3	2	2	0,7
6	4	1	1	0,67
6	4	1	1	0,67
6	4	1	1	0,68
6	4	2	1	0,71
6	4	2	1	0,73
6	4	2	1	0,72
6	4	1	2	0,7
6	4	1	2	0,69
6	4	1	2	0,7
6	4	2	2	0,74
6	4	2	2	0,76
6	4	2	2	0,75
6	5	1	1	0,7
6	5	1	1	0,74
6	5	1	1	0,74
6	5	2	1	0,75
6	5	2	1	0,75
6	5	2	1	0,73
6	5	1	2	0,65
6	5	1	2	0,67
6	5	1	2	0,66
6	5	2	2	0,74
6	5	2	2	0,74
6	5	2	2	0,74
6	6	1	1	0,65
6	6	1	1	0,67
6	6	1	1	0,69
6	6	2	1	0,68
6	6	2	1	0,72
6	6	2	1	0,67
6	6	1	2	0,667
6	6	1	2	0,68
6	6	1	2	0,69
6	6	2	2	0,67
6	6	2	2	0,7

6	6	2	2	0,68
7	1	1	1	0,67
7	1	1	1	0,7
7	1	1	1	0,7
7	1	2	1	0,75
7	1	2	1	0,75
7	1	2	1	0,73
7	1	1	2	0,61
7	1	1	2	0,68
7	1	1	2	0,67
7	1	2	2	0,72
7	1	2	2	0,72
7	1	2	2	0,7
7	2	1	1	0,75
7	2	1	1	0,73
7	2	1	1	0,74
7	2	2	1	0,78
7	2	2	1	0,78
7	2	2	1	0,76
7	2	1	2	0,7
7	2	1	2	0,73
7	2	1	2	0,73
7	2	2	2	0,75
7	2	2	2	0,75
7	2	2	2	0,75
7	3	1	1	0,65
7	3	1	1	0,65
7	3	1	1	0,66
7	3	2	1	0,74
7	3	2	1	0,75
7	3	2	1	0,75
7	3	1	2	0,63
7	3	1	2	0,65
7	3	1	2	0,66
7	3	2	2	0,75
7	3	2	2	0,75
7	3	2	2	0,71
7	4	1	1	0,71
7	4	1	1	0,7
7	4	1	1	0,73
7	4	2	1	0,74
7	4	2	1	0,75
7	4	2	1	0,75
7	4	1	2	0,73
7	4	1	2	0,73
7	4	1	2	0,72
7	4	2	2	0,69
7	4	2	2	0,69

7	4	2	2	0,69
7	5	1	1	0,73
7	5	1	1	0,77
7	5	1	1	0,75
7	5	2	1	0,72
7	5	2	1	0,74
7	5	2	1	0,73
7	5	1	2	0,6
7	5	1	2	0,61
7	5	1	2	0,6
7	5	2	2	0,76
7	5	2	2	0,74
7	5	2	2	0,74
7	6	1	1	0,66
7	6	1	1	0,64
7	6	1	1	0,65
7	6	2	1	0,74
7	6	2	1	0,77
7	6	2	1	0,75
7	6	1	2	0,66
7	6	1	2	0,62
7	6	1	2	0,65
7	6	2	2	0,62
7	6	2	2	0,68
7	6	2	2	0,66
8	1	1	1	0,55
8	1	1	1	0,54
8	1	1	1	0,55
8	1	2	1	0,59
8	1	2	1	0,54
8	1	2	1	0,57
8	1	1	2	0,55
8	1	1	2	0,55
8	1	1	2	0,59
8	1	2	2	0,66
8	1	2	2	0,67
8	1	2	2	0,6
8	2	1	1	0,68
8	2	1	1	0,67
8	2	1	1	0,68
8	2	2	1	0,65
8	2	2	1	0,68
8	2	2	1	0,7
8	2	1	2	0,63
8	2	1	2	0,63
8	2	1	2	0,63
8	2	2	2	0,58
8	2	2	2	0,59

8	2	2	2	0,57
8	3	1	1	0,59
8	3	1	1	0,55
8	3	1	1	0,57
8	3	2	1	0,67
8	3	2	1	0,64
8	3	2	1	0,67
8	3	1	2	0,64
8	3	1	2	0,63
8	3	1	2	0,61
8	3	2	2	0,64
8	3	2	2	0,65
8	3	2	2	0,59
8	4	1	1	0,7
8	4	1	1	0,73
8	4	1	1	0,74
8	4	2	1	0,69
8	4	2	1	0,67
8	4	2	1	0,68
8	4	1	2	0,74
8	4	1	2	0,73
8	4	1	2	0,7
8	4	2	2	0,65
8	4	2	2	0,64
8	4	2	2	0,7
8	5	1	1	0,73
8	5	1	1	0,74
8	5	1	1	0,73
8	5	2	1	0,73
8	5	2	1	0,73
8	5	2	1	0,74
8	5	1	2	0,74
8	5	1	2	0,75
8	5	1	2	0,73
8	5	2	2	0,72
8	5	2	2	0,73
8	5	2	2	0,72
8	6	1	1	0,68
8	6	1	1	0,68
8	6	1	1	0,63
8	6	2	1	0,7
8	6	2	1	0,69
8	6	2	1	0,69
8	6	1	2	0,63
8	6	1	2	0,66
8	6	1	2	0,66
8	6	2	2	0,61
8	6	2	2	0,62

8	6	2	2	0,62
9	1	1	1	0,57
9	1	1	1	0,57
9	1	1	1	0,58
9	1	2	1	0,68
9	1	2	1	0,62
9	1	2	1	0,66
9	1	1	2	0,7
9	1	1	2	0,7
9	1	1	2	0,7
9	1	2	2	0,57
9	1	2	2	0,57
9	1	2	2	0,57
9	2	1	1	0,57
9	2	1	1	0,62
9	2	1	1	0,6
9	2	2	1	0,69
9	2	2	1	0,64
9	2	2	1	0,64
9	2	1	2	0,62
9	2	1	2	0,65
9	2	1	2	0,65
9	2	2	2	0,68
9	2	2	2	0,66
9	2	2	2	0,63
9	3	1	1	0,64
9	3	1	1	0,61
9	3	1	1	0,63
9	3	2	1	0,74
9	3	2	1	0,74
9	3	2	1	0,72
9	3	1	2	0,62
9	3	1	2	0,6
9	3	1	2	0,6
9	3	2	2	0,6
9	3	2	2	0,64
9	3	2	2	0,62
9	4	1	1	0,69
9	4	1	1	0,61
9	4	1	1	0,64
9	4	2	1	0,67
9	4	2	1	0,69
9	4	2	1	0,64
9	4	1	2	0,6
9	4	1	2	0,68
9	4	1	2	0,65
9	4	2	2	0,66
9	4	2	2	0,66

9	4	2	2	0,7
9	5	1	1	0,58
9	5	1	1	0,57
9	5	1	1	0,55
9	5	2	1	0,66
9	5	2	1	0,65
9	5	2	1	0,65
9	5	1	2	0,65
9	5	1	2	0,61
9	5	1	2	0,64
9	5	2	2	0,62
9	5	2	2	0,62
9	5	2	2	0,65
9	6	1	1	0,55
9	6	1	1	0,57
9	6	1	1	0,59
9	6	2	1	0,57
9	6	2	1	0,55
9	6	2	1	0,57
9	6	1	2	0,54
9	6	1	2	0,58
9	6	1	2	0,57
9	6	2	2	0,61
9	6	2	2	0,59
9	6	2	2	0,62
10	1	1	1	0,6
10	1	1	1	0,61
10	1	1	1	0,6
10	1	2	1	0,68
10	1	2	1	0,6
10	1	2	1	0,6
10	1	1	2	0,67
10	1	1	2	0,69
10	1	1	2	0,67
10	1	2	2	0,62
10	1	2	2	0,6
10	1	2	2	0,6
10	2	1	1	0,67
10	2	1	1	0,69
10	2	1	1	0,7
10	2	2	1	0,57
10	2	2	1	0,58
10	2	2	1	0,6
10	2	1	2	0,65
10	2	1	2	0,65
10	2	1	2	0,67
10	2	2	2	0,7
10	2	2	2	0,67

10	2	2	2	0,68
10	3	1	1	0,6
10	3	1	1	0,63
10	3	1	1	0,6
10	3	2	1	0,63
10	3	2	1	0,61
10	3	2	1	0,68
10	3	1	2	0,65
10	3	1	2	0,68
10	3	1	2	0,65
10	3	2	2	0,61
10	3	2	2	0,6
10	3	2	2	0,65
10	4	1	1	0,56
10	4	1	1	0,56
10	4	1	1	0,56
10	4	2	1	0,69
10	4	2	1	0,64
10	4	2	1	0,67
10	4	1	2	0,59
10	4	1	2	0,58
10	4	1	2	0,6
10	4	2	2	0,6
10	4	2	2	0,6
10	4	2	2	0,6
10	5	1	1	0,6
10	5	1	1	0,59
10	5	1	1	0,6
10	5	2	1	0,76
10	5	2	1	0,77
10	5	2	1	0,78
10	5	1	2	0,71
10	5	1	2	0,71
10	5	1	2	0,75
10	5	2	2	0,57
10	5	2	2	0,57
10	5	2	2	0,61
10	6	1	1	0,6
10	6	1	1	0,61
10	6	1	1	0,6
10	6	2	1	0,6
10	6	2	1	0,6
10	6	2	1	0,7
10	6	1	2	0,69
10	6	1	2	0,69
10	6	1	2	0,69
10	6	2	2	0,7
10	6	2	2	0,7

10	6	2	2	0,7
11	1	1	1	0,56
11	1	1	1	0,58
11	1	1	1	0,56
11	1	2	1	0,57
11	1	2	1	0,57
11	1	2	1	0,6
11	1	1	2	0,6
11	1	1	2	0,62
11	1	1	2	0,65
11	1	2	2	0,63
11	1	2	2	0,61
11	1	2	2	0,63
11	2	1	1	0,57
11	2	1	1	0,57
11	2	1	1	0,57
11	2	2	1	0,57
11	2	2	1	0,62
11	2	2	1	0,6
11	2	1	2	0,6
11	2	1	2	0,6
11	2	1	2	0,63
11	2	2	2	0,59
11	2	2	2	0,57
11	2	2	2	0,57
11	3	1	1	0,56
11	3	1	1	0,58
11	3	1	1	0,57
11	3	2	1	0,56
11	3	2	1	0,55
11	3	2	1	0,6
11	3	1	2	0,61
11	3	1	2	0,63
11	3	1	2	0,6
11	3	2	2	0,6
11	3	2	2	0,64
11	3	2	2	0,6
11	4	1	1	0,73
11	4	1	1	0,72
11	4	1	1	0,69
11	4	2	1	0,63
11	4	2	1	0,6
11	4	2	1	0,62
11	4	1	2	0,66
11	4	1	2	0,68
11	4	1	2	0,69
11	4	2	2	0,68
11	4	2	2	0,67

11	4	2	2	0,65
11	5	1	1	0,6
11	5	1	1	0,59
11	5	1	1	0,61
11	5	2	1	0,62
11	5	2	1	0,66
11	5	2	1	0,64
11	5	1	2	0,6
11	5	1	2	0,65
11	5	1	2	0,64
11	5	2	2	0,6
11	5	2	2	0,6
11	5	2	2	0,6
11	6	1	1	0,67
11	6	1	1	0,68
11	6	1	1	0,67
11	6	2	1	0,64
11	6	2	1	0,66
11	6	2	1	0,62
11	6	1	2	0,62
11	6	1	2	0,66
11	6	1	2	0,64
11	6	2	2	0,7
11	6	2	2	0,65
11	6	2	2	0,7
12	1	1	1	0,66
12	1	1	1	0,64
12	1	1	1	0,62
12	1	2	1	0,67
12	1	2	1	0,7
12	1	2	1	0,67
12	1	1	2	0,65
12	1	1	2	0,62
12	1	1	2	0,6
12	1	2	2	0,63
12	1	2	2	0,64
12	1	2	2	0,63
12	2	1	1	0,65
12	2	1	1	0,67
12	2	1	1	0,65
12	2	2	1	0,66
12	2	2	1	0,67
12	2	2	1	0,68
12	2	1	2	0,63
12	2	1	2	0,62
12	2	1	2	0,65
12	2	2	2	0,67
12	2	2	2	0,65

12	2	2	2	0,67
12	3	1	1	0,7
12	3	1	1	0,66
12	3	1	1	0,67
12	3	2	1	0,65
12	3	2	1	0,65
12	3	2	1	0,63
12	3	1	2	0,63
12	3	1	2	0,61
12	3	1	2	0,65
12	3	2	2	0,63
12	3	2	2	0,63
12	3	2	2	0,64
12	4	1	1	0,65
12	4	1	1	0,66
12	4	1	1	0,65
12	4	2	1	0,66
12	4	2	1	0,67
12	4	2	1	0,68
12	4	1	2	0,64
12	4	1	2	0,62
12	4	1	2	0,65
12	4	2	2	0,67
12	4	2	2	0,65
12	4	2	2	0,67
12	5	1	1	0,66
12	5	1	1	0,65
12	5	1	1	0,65
12	5	2	1	0,65
12	5	2	1	0,65
12	5	2	1	0,63
12	5	1	2	0,63
12	5	1	2	0,62
12	5	1	2	0,65
12	5	2	2	0,64
12	5	2	2	0,63
12	5	2	2	0,64
12	6	1	1	0,64
12	6	1	1	0,67
12	6	1	1	0,67
12	6	2	1	0,67
12	6	2	1	0,64
12	6	2	1	0,7
12	6	1	2	0,73
12	6	1	2	0,7
12	6	1	2	0,7
12	6	2	2	0,7
12	6	2	2	0,73

12	6	2	2	0,73
